# Psychometric analysis of the Self-Harm Inventory using Rasch modelling

**DOI:** 10.1186/1471-244X-9-53

**Published:** 2009-08-19

**Authors:** Shane Latimer, Tanya Covic, Steven R Cumming, Alan Tennant

**Affiliations:** 1 School of Psychology, University of Western Sydney, Locked Bag 1797 Penrith South DC 1797 NSW, Australia; 2 Faculty of Health Sciences, University of Sydney, PO Box 170 Lidcombe 1825 NSW, Australia; 3 Faculty of Medicine and Health, Worsley Building, University of Leeds, Leeds LS2 9JT, UK

## Abstract

**Background:**

Deliberate Self-Harm (DSH) is the intentional destruction of healthy body tissue without suicidal intent. DSH behaviours in non-clinical populations vary, and instruments containing a range of behaviours may be more informative than ones with restricted content. The Self-Harm Inventory (SHI) is a widely used measure of DSH in clinical populations (mental and physical health) and covers a broad range of behaviours (self-injury, risk taking and self-defeating acts). The test authors recommend the SHI to screen for Borderline Personality Disorder (BPD) using a cut-off score of five or more. The aim of this study was to investigate the psychometric characteristics of the SHI in non-clinical samples.

**Methods:**

The SHI was administered to a sample of 423 non-clinical participants (university students, age range 17 to 30). External validation was informed by the administration of the Depression Anxiety Stress Scales 21 (DASS-21) to a sub-sample (n = 221). Rasch analysis of the SHI was conducted to provide a stringent test of unidimensionality and to identify the DSH behaviours most likely to be endorsed at each total score.

**Results:**

The SHI showed adequate fit to the Rasch model and no modifications were required following checks of local response dependency, differential item functioning and unidimensionality. The scale identified gender and age differences in scores, with females and older participants reporting higher levels of DSH. SHI scores and DASS-21 scores were related.

**Conclusion:**

The recommended cut-off point of five is likely to comprise mild forms of DSH and may not be indicative of psychopathology in a non-clinical population. Rather it may be more indicative of developmentally related risk taking behaviours while a higher cut-off point may be more suggestive of psychopathology as indicated by higher levels of depression, stress and anxiety.

## Background

Deliberate self-harm (DSH) is the intentional destruction of healthy body tissue without conscious suicidal intent [1] and typically includes behaviours such as cutting, burning, scratching and head banging [2]. However, broader definitions of DSH may include a range of self-harming behaviours [3] from some with no immediate physical tissue damage (i.e. self-starvation or alcohol abuse) [2,4], to those that include suicide-related behaviours (i.e. self-poisoning) [5]. There are a number of theories explaining DSH, including affect regulation, depersonalisation, and behavioural/environmental [6] but there remains a lack of consensus on the aetiology of DSH [7].

The prevalence rates of DSH range between 4% to 20% in adult inpatients and up to 40% in adolescent inpatients [8]. In non-clinical populations, the estimates range between 12% and 66% in high school students [3,9,10], and 12% and 38% in college/university students [11-14]. The highest risk age group for DSH is 18 to 34 years with a female to male ratio estimated at 8:1 for adolescents and at 1.6:1 for the 20 – 50 age group [15].

The great disparity in prevalence estimates for DSH arises in part from a lack of consensus in the conceptualisation of DSH [7,16] and a concomitant diversity in its measurement [17]. Some studies have measured DSH with only one or two items [9,18,19], while others have focused on a limited range of DSH behaviours [20] or have included both suicidal and DSH behaviours (i.e. Self-Harm Behavior Questionnaire [21]). Some studies have used semi-structured [22] or comprehensive interviews (i.e. Suicide Attempt Self-Injury Interview [23]; Deliberate Self-Harm Interview Schedule [24]; Self-Injurious Thoughts and Behaviors Interview [25]) while others have developed self-report scales (i.e. Self-Harm Inventory [4]; Deliberate Self-Harm Inventory [26]).

Studies of DSH in non-clinical populations show variation in the reported forms of DSH [27]. It is suggested that the list of behaviours asked to endorse should be comprehensive to avoid underreporting [28]. The Self-Harm Inventory (SHI) was, therefore, selected for the present study as it includes a broad range of DSH behaviours: non-physical (i.e. self-defeating thoughts) and physical (i.e. cut self), direct (i.e. hit self) and indirect (i.e. abuse alcohol), interpersonal (i.e. be promiscuous) and suicidal (i.e. overdose). Sansone et al [4] developed the SHI based on the conceptualisation of DSH as "...*exists along a continuum from graphic, self-harm behaviour to milder forms of self-sabotaging behaviour that might be viewed as self-defeating*" (p 973) with the specific aim of using self-reports of DSH to diagnose Borderline Personality Disorder (BPD). DSH is one of the diagnostic features of BPD [15] and is commonly present in BPD populations with estimates as high as 75% [29].

The SHI was developed with a sample of 221 participants across three groups: a primary care setting for obesity treatment, a private psychiatric facility for substance abuse and eating disorder treatment, and a family physician for routine health care. Using the Diagnostic Interview for Borderlines (DIB) [30] to diagnose BPD, Sansone et al [4] recommend a cut-off score of 5 on the SHI to provide the best balance between sensitivity (the proportion who have the condition correctly identified by the test) of 88.7% and specificity (the proportion without the condition correctly identified) of 82.1%.

The SHI's convergent validity has been demonstrated [4] by high correlations with the DIB (r = .76, p < .01, n = 221), and with the Personality Diagnostic Questionnaire-Revised [PDQ-R; [31] (r = .73, p < .01, n = 221). As examples of the Cronbach's Alpha values obtained for the SHI, Sansone et al. [32] reported .89 for a sample of 52 women (aged from 24 to 70 years), Sansone et al. [33] reported .90 for a sample of 57 women and 36 males (average age of 41.8 years) and Sansone et al. [34] reported .80 for a sample of 46 males and 61 females (aged between 18 and 65 years). While there is good evidence to support the internal consistency of the scale, no studies have tested the unidimensionality of the SHI. Unfortunately, Cronbach's Alpha does not provide evidence for unidimensionality [35].

Several studies by Sansone and colleagues have utilised the SHI in relation to various conditions such as employment disability [33], domestic violence [36], childhood trauma [37], and suicide attempts [34].

As the SHI includes a broad range of behaviours that may characterise a single latent construct of severity of DSH (i.e. DSH continuum), it is of interest to formally examine this construct. Given that the SHI was developed and mostly used with clinical populations (with mental and physical health problems), it is also important to consider its applicability to a non-clinical population as a risk screening tool for DSH.

The current study will address the following research questions by testing the scale against the requirements of the Rasch measurement model [38] (see Methods for full description) which is based on the Item Response Theory [39] and is increasingly used in the development of new scales and in the improvement of existing scales [40]:

1. Does the SHI meet Rasch model's expectations, its assumptions of unidimensionality, and the stability of responses across age (17–19 year old/20–30 year old), gender (male/female) and mode of administration (pen and paper/online)?

2. Is there evidence for a continuum from mild to severe DSH behaviours as postulated by Sansone et al [4]?

3. Is the cut-off point recommended by Sansone et al [4] meaningful in a non-clinical population?

## Methods

### Participants

The participants were 448 first year Australian university students with secondary school as their highest level of education. There were 365 females and 83 males, with an age range of 17 to 52 years (mean age = 20.61, SD = 5.39). Participants were recruited from two universities. Data were collected via an online survey (n = 301) at one university and via a standard pen and paper survey (n = 147) at the other university. The two universities were from the same large city covering a broad geographical area to capture a wide socioeconomic range.

In order to match the study sample to the age group with the highest rate of DSH [15], 25 participants over the age of 30 were excluded. The reduced sample of 423 comprised 342 females and 81 males (4:1 ratio) with a mean age of 19.45 (SD = 2.14). The online survey group comprised 247 females and 31 males (8:1 ratio) (mean age = 19.75, SD = 2.36) and the pen and paper survey comprised 95 females and 50 males (2:1 ratio) (mean age = 18.86, SD = 1.49). The two modes were not equivalent on age, *t *(421) = 4.14; p = < .001 (two-tailed), and differed on gender ratios.

### Measures

The measures for this study consisted of demographic data (age and gender), the Self-Harm Inventory (SHI) and the Depression, Anxiety and Stress Scales 21 (DASS-21) [41]. The SHI [4] is a 22 item, self-report, yes/no scale that explores a broad range of self-harm behaviours (Table 1). The items are preceded by the statement: '*have you ever intentionally, or on purpose*..." to ensure exclusion of accidental self-harm. Each 'yes' item is counted toward an overall total of behaviours with scores of five and over considered to be indicative of psychopathology and highly correlated with BPD in clinical populations, as demonstrated in the SHI authors' studies [4,42]. The SHI includes one item covering attempted suicide which is outside our accepted definition of DSH [1], however this item has been retained to test the psychometric properties of the complete version of the SHI.

**Table 1:** Fit of Self-Harm Inventory (SHI) Items

SHI Item	Loc.	SE	Fit Res.	DF	Chi Sq.	DF	Prob.
1. Overdosed	0.709	0.173	-2.159	322.62	11.163	6	0.083
2. Cut	-0.686	0.128	-1.935	322.62	12.260	6	0.056
3. Burned	0.891	0.183	-0.444	322.62	5.171	6	0.522
4. Hit Yourself	-1.233	0.122	0.696	322.62	4.119	6	0.661
5. Banged Your Head	-0.892	0.126	-0.408	320.72	6.565	6	0.363
6. Abused Alcohol	-1.793	0.121	2.108	322.62	3.897	6	0.691
7. Driven Recklessly	-0.695	0.129	1.074	321.67	6.945	6	0.326
8. Scratched Yourself	-0.776	0.127	-1.181	321.67	6.410	6	0.379
9. Prevent Wounds Healing	0.265	0.153	-1.149	322.62	4.952	6	0.550
10. Medical Situations Worse	-0.093	0.142	-0.674	322.62	4.077	6	0.666
11. Promiscuous	0.246	0.153	-0.291	322.62	3.706	6	0.716
12. Set Up Relationship Rejection	0.000	0.144	-0.362	322.62	6.810	6	0.339
13. Abused Medication	0.389	0.158	-2.285	322.62	7.547	6	0.273
14. Distanced From God	0.086	0.147	1.116	322.62	6.491	6	0.371
15. Emotional Abuse Relationship	0.088	0.147	-0.952	322.62	4.073	6	0.667
16. Sexual Abuse Relationship	2.990	0.415	-0.773	322.62	2.879	6	0.824
17. Lost Job On Purpose	0.337	0.156	1.910	322.62	18.010	6	0.006
18. Attempted Suicide	0.692	0.172	-1.840	321.67	9.086	6	0.169
19. Exercised Injury	0.217	0.152	-0.191	322.62	4.937	6	0.552
20. Self-Defeating Thoughts	-1.916	0.122	-0.396	321.67	7.067	6	0.315
21. Starved Yourself	-0.414	0.134	-1.279	321.67	6.388	6	0.381
22. Abused Laxatives	1.589	0.232	-0.218	322.62	4.663	6	0.588

The Depression Anxiety Stress Scales 21 (DASS-21) is a short form of Lovibond and Lovibond's [41] 42-item self-report measure of depression, anxiety and stress. It consists of three 7-item subscales that require responses on a 4-point Likert scale, ranging from 0 (*did not apply to me at all*) to 3 (*applied to me very much, or most of the time*). Scores range between 0 and 42 on each subscale. On the depression subscale, scores above 20 indicate severe depression; scores above 14 on the anxiety subscale indicate severe anxiety; and scores above 25 on the stress subscale indicate severe stress. The DASS-21 is widely used and shows good convergent and discriminant validity, as well as high internal consistency and reliability. Cronbach's Alpha has been reported at .88 for Depression subscale, .82 for Anxiety and .90 for Stress [43,44].

### Procedure

Ethics approval was obtained from both universities and the participants received a research participation credit as well as a written debrief and a professional support contact information.

### Data Analysis

Data was analysed using Rasch analysis which tests if a set of summative data meets the rules for constructing interval scale measurement [45]. In the context of health outcome measurement the process is described in detail elsewhere [46]. The process involves a number of activities, which include testing to see if the data meet Rasch model expectations; information on the quality of individual items including individual item fit; testing the assumption of unidimensionality; checking to see if the scale works in the same way across groups (invariance as determined by Differential item Functioning); and examining the reliability and targeting of the scale to the sample.

Briefly, fit to the Rasch model is achieved when a summary chi-square interaction statistic is non-significant, showing no deviation from model expectation; where item and person summary fit statistics show a mean of zero and standard deviation of 1; where individual items show non-significant chi-square fit statistics (Bonferroni adjusted), and where individual item and person residuals are within the range of +/- 2.5. In addition, the scale is expected to show invariance across key groups (e.g. gender or age), as indicated by a non-significant ANOVA of the residuals where group is the main factor, and to demonstrate strict unidimensionality, as indicated by an independent *t-test *on separate estimates for each respondent where less than 5% of such tests should be significant (the separate estimates are derived from subsets of items identified by a principal component analysis of the residuals). Reliability indices are also calculated, namely, Cronbach's Alpha and the Person Separation Index (PSI). The PSI is analogous to Cronbach's Alpha in interpretation but has the advantage of being provided when there are missing cases [47].

The Rasch analysis was conducted using RUMM2020 software [48].

## Results

The score distribution for the 423 cases used for the Rasch analysis showed 62.2% scoring 0 to 4, 30% scoring 5 to 10, and 7.8% scoring 11 or more. There were 84 cases with a score of 0 while for the 339 cases who endorsed at least one DSH behaviour, the average total score was 5.16 (SD = 3.6) (score range 1 to 17).

### Research Question 1: Does the SHI meet Rasch model expectations in terms of unidimensionality and the stability of responses across age (17–19 year old/20–30 year old), gender (male/female) and mode of administration (pen and paper/on-line)?

#### Tests of Fit

The item-trait interaction was non-significant, indicating concordance with model expectations (χ^2 ^= 147.216, df = 132, p = .173). The statistics for the residuals for persons (mean = -0.189, SD = 0.66) were close to the values expected when there is adequate fit to the model (mean = 0, SD = 1). The statistics for the residuals for items (mean = -0.438, SD = 1.213) also supported model fit. The PSI for the SHI was 0.82 and this indicated reasonable person separation reliability and the Cronbach's Alpha was 0.83. All items showed fit to the Rasch model (see Table 1). Fit residual values were all less than the critical value of +/- 2.5. Chi-square probability values were all higher than the Bonferroni adjusted alpha value of 0.002.

#### Differential Item Functioning and Unidimensionality

Differential item functioning was tested for gender, age and mode of administration and with the number of cases in each level of the person factor made equal by random selection from the level with the largest number of cases. There were no significant uniform or non-uniform differences for gender, age or mode of administration using a Bonferroni-adjusted *p *value of 0.0011 (.05/44). There were no correlations above 0.3 in the residual correlations and all items were therefore considered to be free of local response dependency [46]. The final check of dimensionality was conducted using two subtests containing 6 items each with the highest loadings (positive and negative) on a principal component analysis of the residuals. Fourteen (4.13%) of the 339 *t-tests *were significant thereby satisfying the 5% criteria for unidimensionality.

#### Age, Gender and Mode of Administration

Given the absence of item bias in the 22 SHI items, it was appropriate to examine the differences in the scores for age, gender and mode of administration for the 339 participants who endorsed at least one SHI item. Females (n = 272, mean = 5.75, SD = 3.75) reported significantly more DSH than males (n = 67, mean = 4.31, SD = 2.79): *t *(337) = 2.159; p < .05 (two-tailed). The younger age group (17 to 19 years: n = 225, mean = 4.69, SD = 3.34) reported significantly less DSH than the older age group (20 to 30 years: n = 114, mean = 6.08, SD = 3.92): *t *(337) = 3.40; p = < .05 (two-tailed). The web administration group (n = 221, mean = 5.51, SD = 3.86) reported significantly more DSH than the pencil and paper administration group (n = 118, mean = 4.50, SD = 2.97): *t *(337) = 2.283; p < .05 (two-tailed). However, separate *t-test *comparisons for males, females, younger age group and older age group on mode of administration were all non-significant (*p *> .05), suggesting that the higher scores for the web mode were due to the higher proportion of females and the older average age of the participants in the web administration group.

### Research Question 2: Is there evidence for a continuum from mild to graphic DSH behaviours as postulated by Sansone et al [4]?

#### Targeting of Person and Items

**Figure 1:**
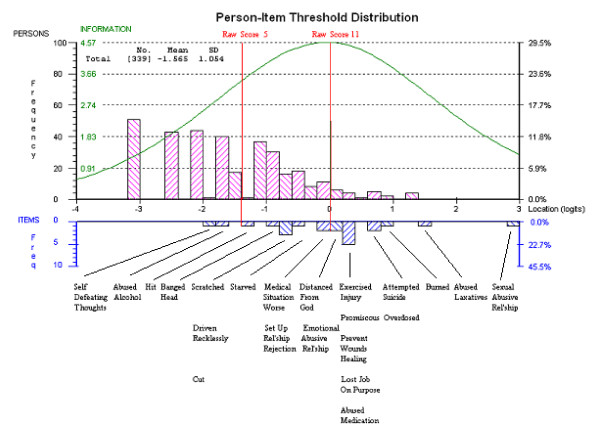
**Distribution of Persons and Item Locations on Common Logit Unit Scale**.

The distributions of item difficulties and person abilities are shown in Figure 1, not including the 84 cases with a raw score of 0. The mean location of persons on the DSH latent trait was -1.566 indicating that the sample, as a whole, exhibited a lower level of DSH behaviour than the average level of DSH measured by the scale. The locations on the latent continuum corresponding to raw scores of 5 (clinical cut-off score) and 11 (peak of test information function) were -1.418 and -0.239 logits, respectively.

#### Dispersion of Items Locations

Figure 1 also shows the dispersion of item locations on the latent continuum ranging from the most easy to endorse (20: *Self-Defeating Thoughts*) to the most difficult to endorse (16: *Sexual Abuse Relationship*). The item with average difficulty (located at 0.0 logits) was 12 (*Set Up Relationship Rejection*). Table 2 shows the observed percentage of the sample endorsing each item (listed from easy to endorse to hard to endorse) as well as the theoretical probabilities of item endorsement for raw score 5 and raw score 11. As clear in Table 2, the logit scale distances between items are not the same as differences in the frequency of item endorsement for the whole sample. For example, the difference in the percentage endorsing *abused medication *and *attempted suicide *is 3% but they differ in location by .30 logits. *Banged your head *and *scratched yourself *also differ by 3% in their raw frequencies but the distance between them is only 0.12 logits.

**Table 2:** Overall Level of Item Endorsement and Theoretical Probabilities of Endorsing Self-Harm Inventory (SHI) Items for Raw Scores 5 and 11

SHI Item	Type	Location	% of Sample Endorsing Item	Theoretical Probability of Item Endorsement at Raw Score 5	Theoretical Probability of Item Endorsement at Raw Score 11
20. Self-Defeating Thoughts	Cognitive	-1.916	55	.62	.87
6. Abused Alcohol	Indirect	-1.793	55	.59	.85
4. Hit Yourself	Direct	-1.233	43	.45	.77
5. Banged Your Head	Direct	-0.892	36	.37	.70
8. Scratched Yourself	Direct	-0.776	33	.35	.68
7. Driven Recklessly	Indirect	-0.695	32	.33	.66
2. Cut	Direct	-0.686	31	.33	.66
21. Starved Yourself	Discreet	-0.414	26	.27	.60
10. Medical Situations Worse	Discreet	-0.093	21	.21	.52
12. Set Up Relationship Rejection	Interpersonal	0.000	20	.20	.49
14. Distanced From God	Interpersonal	0.086	19	.18	.47
15. Emotional Abuse Relationship	Interpersonal	0.088	19	.18	.47
19. Exercised Injury	Discreet	0.217	17	.16	.44
11. Promiscuous	Interpersonal	0.246	17	.16	.43
9. Prevent Wounds Healing	Discreet	0.265	17	.16	.43
17. Lost Job On Purpose	Interpersonal	0.337	17	.15	.41
13. Abused Medication	Discreet	0.389	16	.14	.40
18. Attempted Suicide	Suicidal	0.692	13	.11	.33
1. Overdosed	Suicidal	0.709	13	.11	.32
3. Burned	Direct	0.891	11	.09	.29
22. Abused Laxatives	Discreet	1.589	06	.05	.17
16. Sexual Abuse Relationship	Interpersonal	2.990	02	.01	.05

### Research Question 3: Is the cut-off point recommended by Sansone et al [4] meaningful in a non-clinical population?

The recommended clinical cut-off score on the SHI is five [4]. The dispersion of item locations (Figure 1) means that the expected response pattern for a person at a raw score of 5 is most likely to include items related to cognitive (*self-defeating thoughts*) low level direct (*hitting, banging, scratching*) and indirect (*alcohol abuse, reckless driving*) physical destruction (theoretical probabilities all greater than .30). However, the expected response pattern is less likely to include any of the interpersonal, discreet (medical) and suicidal behaviours (theoretical probabilities all less than .30). Those additional behaviours are more likely to be evident at a raw score of 11 and may be more indicative of psychopathology.

For the purpose of testing the external validity of SHI, a subsample of participants (n = 221) completed DASS-21. DASS-21 scores were compared across three severity levels on the SHI, low (scores 1 to 4, n = 108), medium (scores 5 to 10, n = 85), and high (scores 11 or more, n = 28). In order from low to high SHI severity, mean depression scores were 9.53 (SD = 8.15), 11.58 (SD = 8.60), and 17.00 (SD = 10.51). Mean anxiety scores were 7.09 (SD = 6.53), 11.41 (SD = 8.24), and 16.86 (SD = 11.39). Mean stress scores were 11.67 (SD = 6.40), 16.52 (SD = 9.22), and 23.29 (SD = 9.53). Means differed significantly for depression (F(2, 218) = 8.392, p < .001), for anxiety (F(2, 218) = 18.996, p < .001), and for stress (F(2, 218) = 26.059, P < .001). Post hoc comparisons (using Tukey tests, p < .05) demonstrated that depression differed between the low and high SHI levels and between the medium and high SHI levels, but not between low and medium levels. Anxiety and stress means differed significantly in all three pairwise comparisons.

## Discussion

The aim of this study was to use Rasch analysis to test the internal validity of the Self-Harm Inventory (SHI) scale in a non-clinical population. In terms of the gender differences, females reported higher levels of DSH than males, which is consistent with other studies [22]. Older participants reported higher rates of DSH than younger participants, as noted by others [13]. While other research has reported greater self-disclosure associated with the web-based mode of administration [49], the higher DSH scores for the web-based mode in this study were due to the higher proportion of females and the older age of the web-based participants. Some research [50] suggests that the mode of administration may alter the construct being measured. The lack of differential item functioning for mode of delivery in this study suggests that the nature of the latent construct appears to be consistent across methods of administration.

The ordering of the items ranged from the easiest to endorse (least severe), *self-defeating thoughts*, to the most difficult to endorse (most severe), *sexually abusive relationships*, with a progression across physically dangerous DSH behaviours such as *cutting *to *suicide attempts*, *overdosing *and *burning*. The item ordering accounted for both the experimental, risk taking behaviours commonly seen in adolescents (i.e. alcohol abuse, driving recklessly) [2,51] as well as more clinically significant psychopathologies (i.e. starving, overdosing) [52,53].

This progression of behaviours may characterise a single latent construct of severity of DSH (as supported by the tests of unidimensionality) which in turn may provide support for a continuum conceptualisation of DSH [2]. However, as asserted by Bejar [54]: "*unidimensionality does not imply that performance on the items is due to a single psychological process*" but may result from several psychological processes "*as long as they function in unison*" (p. 31). Therefore, responses on the SHI may relate to different psychological processes, at least in this study's non-clinical population. Exploration of those psychological processes was outside of the scope of this study, as was a comparison of non-clinical to clinical populations.

Our findings suggest, in the absence of clinical verification, that a raw score of five is most likely to comprise milder forms of DSH such as *hit, bang *and *scratch *plus engage in *self-defeating thoughts *and *alcohol abuse *rather than more serious direct physical DSH and interpersonal and suicidal behaviours. Hitting and scratching are reported as one of the most common forms of DSH [8,22,55], while self-defeating thoughts are common negative cognitions that, outside of a pattern of problematic behaviours, would not be considered as DSH. Cutting, which is the most common DSH behaviour [9,11,56-58], was shown in our study to be more difficult to endorse than *hit, bang *and *scratch *which suggests that this behaviour may be a useful marker for the progression from milder to more severe forms of DSH.

Sansone et al. [59] have reported that individuals with BPD, as confirmed by clinical interviews, are likely to endorse the more serious forms of DSH such as *cut*, *overdosed*, *burned *and *attempted suicide*, and obtain a total score more in the vicinity of 10 or 11. We have found a similar pattern of behaviours at the score of 11 but not at the recommended cut-off of five. Therefore, a score of five in a non-clinical population may not be indicative of psychopathology but a score of 11 may be suggestive of some psychopathology. The scale authors in their studies with clinical populations have found these cut-off scores to be correlated with a diagnosis of BPD. Although we have not measured BPD in our study, this level of association is unlikely in a non-clinical population as almost 8% of our sample scored 11 and above, which is considerably higher than the 2% prevalence rate of BPD in community [60].

Our study supports the use of the SHI in a non-clinical population to provide an informative profile of overt and covert behaviours that may identify those at risk of psychopathology other than BPD. The levels of depression, anxiety and stress were significantly elevated for participants with high scores on the SHI. This result is consistent with Klonsky and Olino's [61] study of a non-clinical sample in which the highest level of psychological symptoms (also measured on the DASS-21) were reported by participants engaging in the widest range of DSH. The specific finding for depression (i.e. significant increase above raw score 11 but not above raw score 5) suggests that psychological wellbeing may be at particular risk when individuals report a range of DSH behaviours (as evident in the SHI response patterns for scores around 11).

Based on the Rasch analysis, the SHI may be improved by constructing more items at the less severe end of the latent construct to reduce the significant floor effect. Also, it may be possible to reduce the number of items located around the average item difficulty. Further, more items are needed at the severe end of the latent construct to measure the possible chronic DSH behaviours located beyond suicide related DSH. One of the two extreme items (*abused laxatives*) suggests that eating disorder may be one of the chronic behaviours. The DSH construct indicated by the other extreme item (*sexual abusive relationships*) is not clear.

A number of limitations need to be acknowledged. The university sample may not be representative of the larger community although it comes from geographically and economically broad areas as covered by two universities from the same city; the non-clinical status assumption was not clinically validated (however, the use of DASS-21 provided mood status indication); and other DSH or related constructs' scales were not included to illuminate the external validity of this scale. However, while there are numerous DSH scales, only preliminary psychometric evaluations have been conducted and as such there is no 'gold standard' scale to use as an independent measure. Also while the male population was under-represented in this sample, the gender sub-samples met the statistical size requirements.

## Conclusion

Notwithstanding these limitations, this study provides the first stringent evaluation of one of the self-report scales of DSH using Rasch analysis. Further, such evaluations of the SHI and other DSH scales and across clinical and non-clinical populations may lead to a standardised measure of DSH. This will aid research by providing a clearer conceptualisation of DSH, and clinical practice by providing an empirically validated severity scale that, for example, may identify the DSH behaviours most likely to indicate transition to more serious DSH.

## Competing interests

The authors declare that they have no competing interests.

## Authors' contributions

TC and SRC participated in the study design and coordination. SL, TC and AT performed the statistical analysis. SL and TC drafted the manuscript. All authors contributed to and approved the final manuscript.

## Pre-publication history

The pre-publication history for this paper can be accessed here:


